# Dynamic confinement of SAPO-17 cages on the selectivity control of syngas conversion

**DOI:** 10.1093/nsr/nwac146

**Published:** 2022-07-26

**Authors:** Haodi Wang, Feng Jiao, Yi Ding, Wenjuan Liu, Zhaochao Xu, Xiulian Pan, Xinhe Bao

**Affiliations:** State Key Laboratory of Catalysis, 2011-Collaborative Innovation Center of Chemistry for Energy Materials, Dalian Institute of Chemical Physics, Chinese Academy of Sciences, Dalian 116023, China; University of Chinese Academy of Sciences, Beijing 100049, China; State Key Laboratory of Catalysis, 2011-Collaborative Innovation Center of Chemistry for Energy Materials, Dalian Institute of Chemical Physics, Chinese Academy of Sciences, Dalian 116023, China; University of Chinese Academy of Sciences, Beijing 100049, China; State Key Laboratory of Catalysis, 2011-Collaborative Innovation Center of Chemistry for Energy Materials, Dalian Institute of Chemical Physics, Chinese Academy of Sciences, Dalian 116023, China; Department of Chemical Physics, University of Science and Technology of China, Hefei 230026, China; Key Laboratory of Separation Science for Analytical Chemistry, Dalian Institute of Chemical Physics, Chinese Academy of Sciences, Dalian 116023, China; University of Chinese Academy of Sciences, Beijing 100049, China; Key Laboratory of Separation Science for Analytical Chemistry, Dalian Institute of Chemical Physics, Chinese Academy of Sciences, Dalian 116023, China; State Key Laboratory of Catalysis, 2011-Collaborative Innovation Center of Chemistry for Energy Materials, Dalian Institute of Chemical Physics, Chinese Academy of Sciences, Dalian 116023, China; University of Chinese Academy of Sciences, Beijing 100049, China; State Key Laboratory of Catalysis, 2011-Collaborative Innovation Center of Chemistry for Energy Materials, Dalian Institute of Chemical Physics, Chinese Academy of Sciences, Dalian 116023, China

**Keywords:** syngas conversion, OXZEO, dynamic confinement, zeolite, diffusion

## Abstract

The OXZEO (oxide−zeolite) bifunctional catalyst concept has enabled selective syngas conversion to a series of value-added chemicals and fuels such as light olefins, aromatics and gasoline. Herein we report for the first time a dynamic confinement of SAPO-17 cages on the selectivity control of syngas conversion observed during an induction period. Structured illumination microscopy, intelligent gravimetric analysis, UV-Raman, X-ray diffraction, thermogravimetry and gas chromatography-mass spectrometer analysis indicate that this is attributed to the evolution of carbonaceous species as the reaction proceeds, which gradually reduces the effective space inside the cage. Consequently, the diffusion of molecules is hindered and the hindering is much more prominent for larger molecules such as C_4+_. As a result, the selectivity of ethylene is enhanced whereas that of C_4+_ is suppressed. Beyond the induction period, the product selectivity levels off. For instance, ethylene selectivity levels off at 44% and propylene selectivity at 31%, as well as CO conversion at 27%. The findings here bring a new fundamental understanding that will guide further development of selective catalysts for olefin synthesis based on the OXZEO concept.

## Teaser

This work reports for the first time a dynamic confinement of SAPO-17 cages on the selectivity control of syngas conversion observed during an induction period.

## INTRODUCTION

Syngas conversion, as the core technology for efficient and clean utilization of carbon resources such as coal, natural gas, CO_2_ and biomass, has received extensive attention from both academia and industry [1–4]. Fischer-Tropsch synthesis (FTS) has been well developed for direct conversion of syngas to liquid fuels ever since it was invented in the 1920s. An increasing number of studies demonstrate that the OXZEO (oxide–zeolite) bifunctional catalyst concept provides an alternative direct syngas conversion technology. It offers an effective strategy to tackle the selectivity challenge encountered in the conventional FTS process [3,5–8]. For instance, by combining SAPO-34 zeotype and ZnCrO_x_ oxides, syngas can be directly converted to mixed light olefins, that is, olefins containing two to four carbon atoms (C_2_^=^–C_4_^=^), with the selectivity up to 80% [3]. The C_2_–C_4_ selectivity, including olefins and paraffins, reaches >90%, which surpasses by a large margin the theoretical limit of 58% predicted by the Anderson-Schulz-Flory distribution model [1].

The high selectivity of the OXZEO process can be attributed to the disconnected CO/H_2_ activation and C-C bond coupling on the two spatially separated active sites, i.e. partially reduced metal oxides and zeolites (zeotypes). Thus, the product selectivity can be modulated by the shape selective zeolitic pores with different acidities and topologies [9,10]. For instance, SAPO-34 [3,8,11,12], SSZ-13 [13], SAPO-18 [7,14] and SAPO-17 [15], with eight member ring (MR) pores, allowed selective synthesis of mixed light olefins. The 8-MR side pocket of mordenite zeolite (MOR), which is smaller than the CHA-cage, favored ethylene formation [6,16]. In comparison, zeolites/zeotypes with 10-MR and 12-MR pores such as ZSM-5, SAPO-11, ZSM-11, ZSM-22 and ZSM-12, favored formation of gasoline ranged hydrocarbons (C_5_–C_11_) [5,17]. Interestingly, the channel structure can further fine-tune the hydrocarbon distribution for gasoline. For instance, one-dimensional 10-MR channels preferred isoparaffin formation, whereas those with three-dimensional 10-MR and 12-MR channels with larger intersections favored the production of aromatics. Furthermore, the morphology of zeolites, which frequently changes the path of molecular diffusion, also has effects on product distribution. For instance, sheet-like ZSM-5, with a lower length ratio of the *b/a* axes, favored formation of aromatics [18]. However, all the above studies explored the shape selectivity and confinement of zeolites during steady-state reactions.

We report for the first time the induction period in syngas conversion over an OXZEO composite catalyst, ZnCrO_x_-SAPO-17. During this period, a dynamic confinement develops within the SAPO-17 cage, which controls the product selectivity due to the evolution of carbonaceous species as the reaction proceeds. As a result, the selectivity of ethylene is enhanced whereas that of C_4+_ hydrocarbons is suppressed.

## RESULTS AND DISCUSSION

### Physicochemical property of SAPO-17

SAPO-17 samples with different Si/Al ratios were synthesized using the hydrothermal method. The resulting samples were named SAPO-17_x_, with x denoting the Si/Al ratio, which was measured to be in the range of 0.05 to 0.19 by X-ray fluorescence (XRF). X-ray diffraction (XRD) confirms the ERI topology (Supplementary Fig. S1). Scanning electron microscopy (SEM) shows that SAPO-17 has a rod-like morphology, ∼2 μm in length (Supplementary Fig. S2). N_2_ physical adsorption and desorption indicates that all samples contain micropores with a surface area ranging from 317.5 to 572.9 m^2^/g and pore volume between 0.125 and 0.223 cm^3^/g (Supplementary Table S1). Ammonia temperature programmed desorption (NH_3_-TPD) and fourier transform infrared spectroscopy (FT-IR) both reveal that all SAPO-17 samples contain medium-strength Brønsted acid sites (Supplementary Fig. S3), which are generally considered as the active sites for C–C coupling [3,8,14].

### Induction period in syngas conversion

**Figure 1.**
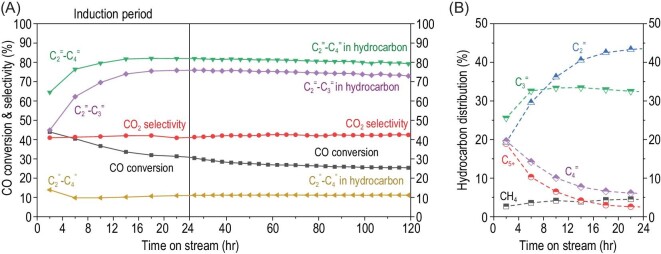
Catalytic performance of syngas conversion over ZnCrO_x_-SAPO-17_0.10_. (A) Dynamic evolution of performance with time on stream. (B) Hydrocarbon distribution during the induction period. Reaction conditions: 400°C, 4.0 MPa, H_2_/CO = 2.5, ZnCrO_x_/SAPO-17 = 1/1 (mass ratio), GHSV = 5000 mL·g^–1^·h^–1^.

Figure 1A shows that the composite ZnCrO_x_-SAPO-17_0.10_ catalyst gives an initial CO conversion of 45% in syngas conversion under 400ºC, 4 MPa and 5000 mL·g^–1^·h^–1^. CO conversion gradually decreases to 31% after reaction for 22 h and then levels off at around 27% after 60 h. At the same time, light olefins (C_2_^=^–C_4_^=^) selectivity increases from the initial 64% to 82%, while that of the more valuable C_2_^=^–C_3_^=^ stabilizes around 75% after the induction period. Note that the hydrocarbon selectivities are reported as CO_2_-free selectivities to simplify the discussion, since CO_2_ remains almost unchanged during the reaction in this study. CH_4_ selectivity stays almost constant throughout the reaction, confirming again that CH_4_ is the primary hydrogenation product over metal oxides in the OXZEO process [3,14]. Figure 1B shows that the ethylene selectivity rises from 19% continuously to 44%, that of C_3_^=^ increases in the first 5 h and then remains almost unchanged, and those of C_4_^=^ and C_5+_ hydrocarbons drop continuously during the induction period.

Supplementary Fig. S4 shows that CO conversion increases with increasing reaction temperature, e.g. reaching 40% at 420°C. However, there is no obvious change in product distribution. A higher gas hourly space velocity (GHSV) from 1000 to 10 000 mL·g^–1^·h^–1^ enhances the light olefins selectivity from 71% to 84% but at the expense of CO conversion, which decreases from 64% to 17% (Supplementary Fig. S5). A higher pressure is also beneficial to enhance CO conversion and does not affect the product selectivity much (Supplementary Fig. S6). Supplementary Fig. S7 displays the catalytic behavior of a series of SAPO-17**_x_** with a varying Si/Al ratio. They all give an obvious induction period, during which the evolution of product selectivities follows a similar trend as in Fig. 1B. Therefore, the induction period is ubiquitous for ZnCrO_x_-SAPO-17 with varying Si/Al ratios.

### Dynamic evolution of confined carbonaceous species

The crystal size of spinel ZnCrO_x_ does not change with the reaction time and remains ∼6.1–6.6 nm, indicating that the oxide is rather stable under this condition (Supplementary Fig. S8 and Supplementary Table S2). Furthermore, there is hardly any Zn detected on SAPO-17 after reaction for 2 and 22 h (Supplementary Fig. S9), indicating that migration of Zn species is negligible here. Therefore, the product selectivity evolution during the induction period is not attributed to ZnCrO_x_ oxide but to SAPO-17.

After reaction for 2 h, the ethylene selectivity is 19% over SAPO-17 and 8% over SAPO-34 (Supplementary Fig. S10). This also reflects the shape selectivity for ethylene formation due to a smaller supercage size of ERI zeolite than that of CHA, similar to that reported previously [19,20]. However, the ethylene selectivity increases significantly up to 44% after the induction period over SAPO-17 (ERI). The corresponding ratio of ethylene to propylene increases to 1.38 in comparison to 0.75 at 2 h. It is also higher than that reported for SAPO-17 in the methanol to hydrocarbon (MTH) process [20]. In comparison, the ethylene selectivity and ratio of ethylene/propylene does not change over ZnCrO_x_-SAPO-34 with a time on stream of 20 h under the same conditions. These results indicate that the selectivity evolution during the induction period over ZnCrO_x_-SAPO-17 is not just attributed to the shape selectivity of the window size or cage size of SAPO-17 (Supplementary Fig. S10). To elucidate the mechanism that controls the product selectivity over SAPO-17 during the induction period, we carefully sorted out SAPO-17_0.10_ granules from the physically mixed composites and analyzed the physicochemical properties of the used SAPO-17 samples as a function of reaction time.

The XRD patterns in Supplementary Fig. S11 show that the ERI topology remains unchanged during the induction period. They further support that the evolution of selectivity is beyond just zeolite-topology-controlled product shape selectivity. IR and NH_3_-TPD in Supplementary Figs S12 and S13 demonstrate that the medium-strength Brønsted acid density remains almost unchanged after the initial 2 h. The IR spectra of SAPO-17 upon pyridine adsorption in Supplementary Fig. S14 confirm that the remaining Brønsted acid sites are mainly located inside the micropores. It is worth noting that NH_3_ desorption shifts toward a higher temperature for SAPO-17 upon a longer reaction time (Supplementary Fig. S13). This is either caused by strengthened acidity or suppressed diffusion rate [21,22]. However, a number of studies have demonstrated that stronger acidity would have facilitated hydrogenation, leading to paraffin formation. Therefore, the increasing light olefins selectivity during the induction period (Fig. 1) cannot be attributed to the zeotype acidity.

**Figure 2.**
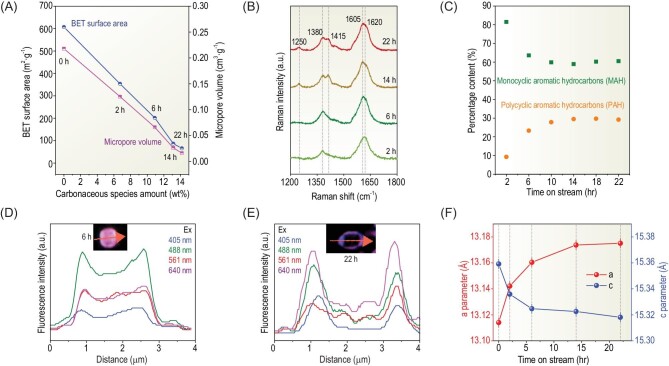
Characterization of the used SAPO-17 during the induction period. (A) Correlation of BET surface area and micropore volume with carbonaceous species. (B) UV-Raman spectra. (C) Monocyclic and polycyclic aromatic hydrocarbons analyzed by GC-MS. (D and E) SIM measurement of the spatiotemporal distribution of carbonaceous species over SAPO-17 after reaction for 6 h and 22 h, respectively. (F) Variation of the unit cell parameters.

Further thermogravimetry (TG) analysis indicates that carbonaceous species accumulate gradually inside the SAPO-17 cages as the reaction proceeds (Supplementary Fig. S15). The weight does not increase further after reaction for 22 h. Correspondingly, the N_2_ adsorption-desorption shows that the Brunauer-Emmett-Teller (BET) surface area drops dramatically from over 600 m^2^·g^–1^ down to ∼50 m^2^·g^–1^ and the micropore volume drops to only 0.02 cm^3^·g^–1^ after reaction for 22 h (Fig. 2A). Interestingly, this does not deactivate the catalyst because the activity and product selectivity almost level off after 22 h (Fig. 1A). Therefore, the Brønsted acid sites should still be accessible to reactants and hence the reaction inside the SAPO-17 cage. Compared to the initial product distribution, the catalyst at this point obviously benefits formation of ethylene.

UV-Raman spectra in Fig. 2B show bands at 1250, 1380 and 1620 cm^–1^, which are assigned to methylbenzene and naphthalene. In addition, the band at 1620 cm^–1^ gradually shifts to 1605 cm^–1^ along with a new band around 1415 cm^–1^ after reaction for 6 h, indicating the formation of more condensed methyl anthracene [23,24]. It is further confirmed by gas chromatography-mass spectrometer (GC-MS) and structured illumination microscopy (SIM) in Fig. 2C–E and Supplementary Figs S16 and S22. These results demonstrate that carbonaceous species evolve from lighter monocyclic aromatic hydrocarbons (MAHs, e.g. durene with a cross-section size of 6.9 × 5.4 Å) to polycyclic aromatic hydrocarbons (PAHs, e.g. tetramethyl-naphthalene of 7.3 × 6.9 Å) as the reaction proceeds [23]. As a result, the free space inside the cage is decreased gradually. Furthermore, the refinement of the XRD patterns reveals that the crystal lattice is distorted due to the accumulated carbonaceous species (Fig. 2F and Supplementary Fig. S23). The *a* axis is enlarged and the *c* axis is shrunk stepwise with the reaction time on stream. A distorted lattice due to carbon deposition inside ZSM-5 cages was also observed previously during MTH [25]. The distortion slows down as the induction approaches completion. This results in a dynamic confinement environment for molecules and their reaction.

### Effective space coefficient and its effects on molecular diffusion

To further understand the dynamic confinement effects on the performance of syngas conversion, we define a descriptor to describe the effective space inside the SAPO-17 cage, namely the ‘effective space coefficient’ (ESC). Assuming the micropores are filled completely with carbonaceous species, extrapolation of the micropore volume curve in Fig. 2A to zero gives a weight percentage of 15 wt%. Thus, the percentage of micropores that are occupied by carbonaceous species at the reaction time (t), is estimated according to:

(1) \begin{equation*} {{{E\!}}}_{{t}} = {{{{{{W}}}_{{t}}}} /{{15}}} \times {100\% }, \end{equation*}

where ${W}_{{t}}$ represents the actual weight percentage of carbonaceous species, measured by TG. Thus, the

free micropores are estimated by:

(2) \begin{equation*} {{{F\!}}}_{{t}} = ({1} - {{{E}}}_{{t}}) \times {100\% }, \end{equation*}

The composition of the carbonaceous species is analyzed by GC-MS **(**Supplementary Fig. S16).

We take the GC-MS signal intensity, ${P}_{\boldsymbol{i}}$, to represent the concentration of molecule *i*, and then the ESC at the reaction time *t* is expressed as:

(3) \begin{eqnarray*} {{E\!S\!C}}_{{t}} &=& \left[ {{{F\!}}}_{{t}} + {{{E\!}}}_{{t}} \times \sum\limits_i {\left[ {\left( {{1} - {{{{{{A}}}_{{i}}}}/{{{A}}}}} \right) \times {{{P}}}_{{i}}} \right]}\right.\nonumber\\ && \times\, {100\% }, \end{eqnarray*}

where *A* represents the projected area of the SAPO-17 cage along the *a*-axis orientation (14.8 × 10.1 Å) and *A_i_* represents the projected area of molecule *i*. It is assumed that molecule *i* takes up the space of ${{{{A}_i}}/{A}}$ inside the cage (Supplementary Tables S3 and S4). A higher ESC reflects more free space inside the SAPO-17 cage and vice versa.

**Figure 3.**
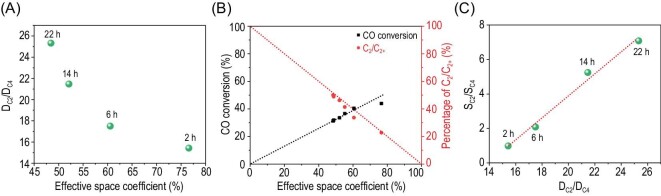
Effects of the dynamic confinement of the SAPO-17 cage on molecular diffusion and catalytic performance. (A) Relation between D_C2_/D_C4_ and ESC*.* (B) The catalytic performance of ZnCrO_x_-SAPO-17 as a function of ESC. (C) Relation between the selectivity of S_C2_/S_C4_ and the diffusion of D_C2_/D_C4_. Reaction conditions: 400°C, 4.0 MPa, H_2_/CO = 2.5, ZnCrO_x_/SAPO-17 = 1/1 (mass ratio), GHSV = 5000 mL·g^–1^·h^–1^.

Interestingly, the SIM images in Fig. 2D and E show that more carbonaceous species are located at the rim region of SAPO-17 crystals. This could create additional obstacles for diffusion of the intermediates and primary products, and thus inhibit further chain growth reaction. Thus, we chose ethane, propane and butane as probe molecules and studied their diffusion by intelligent gravimetric analysis (IGA), which has been frequently employed to study the diffusion of guest molecules inside porous materials [26,27]. As shown in Supplementary Fig. S24, the uptake of these molecules in SAPO-17 decreases with prolonged reaction, suggesting hindered diffusion [28–30]. The hindered diffusion could contribute to the stepwise upshifted NH_3_-TPD peaks to a higher temperature in Supplementary Fig. S13. The diffusion coefficient, D, of different molecules can be estimated according to Fick's second law (Supplementary Equations S1 and S2), as listed in Supplementary Table S5. Figure 3A shows that the diffusion coefficient ratio of C_2_ to C_4_ (denoted as D_C2_/D_C4_) is correlated negatively with ESC, implying more vigorously hindered diffusion for C_4_ than for C_2_, with the reduced free space within the cage. On the other hand, a more spacious cage favors the secondary reaction of ethylene, which would lead to an enhanced CO conversion by shifting the reaction equilibrium of CO conversion to ketene. Thus, the percentage of C_2_/C_2+_ in the product will be lowered, which is supported by the data in Fig. 3B. Furthermore, it is interesting to note an almost linear correlation between the selectivity ratio and diffusion coefficient ratio, i.e. S_C2_/S_C4_ vs. D_C2_/D_C4_ and S_C2_/S_C3_ vs. D_C2_/D_C3_ in Fig. 3C and Supplementary Fig. S25, respectively. This reveals the significant effect of the dynamic confinement of the SAPO-17 cage on molecular diffusion and hence product selectivity.

## CONCLUSION

We report for the first time an induction period in syngas conversion catalyzed by a ZnCrO_x_-SAPO-17 bifunctional catalyst, where SAPO-17 cages exert a dynamic confinement effect on product selectivity. Ethylene selectivity increases from 19% up to 44%, whereas C_4+_ hydrocarbon selectivity declines from 39% to 9% with time on stream. This is attributed to the evolution of carbonaceous species from small hydrocarbon molecules to polycyclic aromatics, evidenced by TG, GC-MS, XRD, UV-Raman and SIM. It leads to a stepwise reduced free space within the cage. IGA indicates that this hinders the diffusion of molecules, particularly larger molecules. The significantly restricted space also suppresses the secondary reaction of ethylene, resulting in an enhanced ethylene selectivity and suppressed formation of higher hydrocarbons. Although the microporous volume is almost completely occupied (93%) at the end of the induction period, this does not deactivate the catalyst. CO conversion levels off at 27%, and C_2_^=^–C_3_^=^ selectivities at 75%. This dynamic confinement is expected to be general for a number of reactions involving hydrocarbons over zeolites. The fundamental understanding is essential for the further design of high-performance zeolite-based catalysts for C1 chemistry as well as other reactions involving hydrocarbons.

## MATERIALS AND METHODS

ZnCrO_x_ oxide was prepared following the same procedure reported previously [3]. SAPO-17 was prepared by the hydrothermal method. The composition of the precursor gel was 1.0 Al_2_O_3_ : 1.0 P_2_O_5_ : (0.06–0.20) SiO_2_ : 1.0 CHA : 50 H_2_O. The mixture was crystallized at 180°C for 72 h under rotation. The product was calcined at 600°C for 6 h.

SEM images were collected by Quanta 200. XRD patterns were recorded on PANAlytical X’pert Pro-1 and a PANAlytical Empyrean diffractometer with PIXcel3D detector. XRF was conducted on a PANAlytical Zetium XRF spectrometer. Nitrogen adsorption-desorption isotherms were obtained on a Quantachrome NOVA 4200e instrument. NH_3_-TPD was performed on a Micromeritics AutoChem 2920 instrument equipped with a thermal conductivity detector (TCD). TG analysis was carried out on an STA 449 F3 instrument. GC-MS was acquired using Agilent 8890–7250 equipped with an HP-5 capillary column and a flame ionization detector (FID). The FT-IR experiment was carried out on a BRUKER TENSOR 27 equipped with an mercury cadmium telluride (MCT) detector. UV-Raman spectra were collected using a home-built spectrometer [24] with a 266 nm constant-wave laser, a 25-mm-diameter off-axis parabolic mirror as the light-collecting element, an edge filter to filter the Rayleigh scattered light, a spectrograph, and a ultraviolet-charge coupled device (UV-CCD) camera produced by Andor. SIM was carried out on a Nikon N-SIM super-resolution microscopy system with a motorized inverted microscopy ECLIPSE Ti2-E, a ×100/numerical aperture 1.49 oil-immersion total internal reflection fluorescence objective lens (CFI HP) and an ORCA-Flash 4.0 sCMOS camera (Hamamatsu Photonics K.K.). The uptake rates of ethane, propane and *n*-butane were measured by IGA100 of Hiden Analytical.

Syngas conversion was performed in a continuous-flow fixed-bed stainless steel reactor furnished with a quartz lining. Products were analyzed by an online gas chromatography (GC) (Agilent 7890B) equipped with a TCD and an FID. Hayesep Q and 5 Å molecular-sieve-packed columns were connected to the TCD while HP-FFAP and HP-AL/S capillary columns were connected to the FID.

## Supplementary Material

nwac146_Supplemental_File
