# Supplementary material for: Dynamic confinement of SAPO-17 cages on the selectivity control of syngas conversion
Source: Natl Sci Rev. 2022 Jul 26;9(9):nwac146. doi: 10.1093/nsr/nwac146 (PMC9477199; doi:10.1093/nsr/nwac146)
Supplement: nwac146_Supplemental_File [file nwac146_supplemental_file.pdf]

Supporting Information

Haodi Wang[1,3], Feng Jiao[1,3,*], Yi Ding[1,4], Wenjuan Liu[2,3], Zhaochao Xu[2], Xiulian Pan[1,3,*], and Xinhe Bao[1,*]

[1] State Key Laboratory of Catalysis, 2011-Collaborative Innovation Center of Chemistry for Energy Materials, Dalian Institute of Chemical Physics, Chinese Academy of Sciences, Dalian 116023, (China).

[2] Key Laboratory of Separation Science for Analytical Chemistry, Dalian Institute of Chemical Physics, Chinese Academy of Sciences, Dalian 116023, (China).

[3] University of Chinese Academy of Sciences, Beijing 100049, (China).

[4] Department of Chemical Physics, University of Science and Technology of China, Hefei 230026, (P. R. China).

***Corresponding authors**. E-mails: jiaofeng@dicp.ac.cn; panxl@dicp.ac.cn; xhbao@dicp.ac.cn

Experimental Section

Catalyst preparation

$ZnCrO_x$ oxide was prepared following the same procedure reported previously [1].

SAPO-17 was prepared by the hydrothermal method following a previously reported procedure [2]. Typically, aluminum isopropoxide was first dissolved in deionized water, then phosphoric acid (85% by mass), silica sol (30% by mass) and cyclohexylamine were added in sequence. The composition of the gel was 1.0 Al_2O_3: 1.0 P_2O_5: (0.06-0.20) SiO_2: 1.0 CHA: 50 H_2O. The mixture was transferred to a stainless steel autoclave lined with polytetrafluoroethylene and crystallized at 180°C for 72 h under rotation. After centrifugation, washing and drying, the product was calcined at 600°C for 6 h. The resulting samples were named as SAPO-17_x (x = 0.06-0.20).

Characterization

The morphology was characterized by Quanta 200 Scanning Electron Microscope (SEM).

X-ray diffraction (XRD) patterns were recorded on PANAlytical X'pert Pro-1 equipped with Cu Kα radiation (λ = 1.5418 Å), operated at 40 kV and 40 mA. For Rietveld refinement, high resolution powder X-ray diffraction (PXRD) was performed on PANAlytical Empyrean diffractometer equipped with Cu Kα X-ray (λ = 1.54056 Å) tubes with PIXcel3D detector. PXRD patterns were recorded in the range of 2θ = 5-120°, using a step size of 0.026° and scan step time of 397.29 s. All data were carefully analyzed using HighScore Plus 4.6 by Pawley method.

The element composition of the samples was determined on a PANAlytical Zetium X-Ray Fluorescence (XRF) spectrometer.

Nitrogen adsorption-desorption isotherms were obtained at -196°C on a Quantachrome NOVA 4200e instrument. The sample was pretreated under vacuum at 300°C for 6 h. The specific surface area and pore volume were calculated based on Brunauer-Emmett-Teller (BET) and t-plot method, respectively.

Temperature Programmed Desorption of NH_3 (NH_3-TPD) was performed on a Micromeritics AutoChem 2920 instrument equipped with a thermal conductivity detector (TCD). Typically, 100 mg sample was pretreated under a flowing Ar at 450°C for 1.5 h. After cooling down to 100°C, the sample was exposed to 5% NH_3/He for 30 min followed by sweeping in He until a stable baseline was obtained. Subsequently, the TCD signal was recorded with the temperature increased from 100 to 800°C at a heating rate of 10°C/min. For the used SAPO-17 sample, the same procedure was followed except that 50 mg was used. The sign of m/z=17 was recorded by an online mass spectrometer.

Thermogravimetric (TG) analysis was proceeded on an STA 449 F3 instrument at a heating rate of 10°C/min from 40 to 950℃ under flowing air (20 mL/min).

The carbonaceous species were analyzed by a gas chromatography-mass spectrometry (GC-MS) (8890-7250) equipped with an HP-5 capillary column and a flame ionization detector (FID). Typically, 50 mg used catalyst was dissolved in 2.0 mL 10% HF solution and extracted with 2.0 mL CH_2Cl_2.

Fourier Transform Infrared Spectroscopy (FT-IR) experiment was carried out on a BRUKER TENSOR 27 equipped with an MCT detector. The sample was pressed into a wafer and placed inside an *in-situ* quartz cell. Then it was dehydrated ($<10^{-2}$ Pa) at 450°C for 3 h. After cooling down to room temperature, the spectra were taken by averaging 64 scans collected at 4 cm^{-1} resolutions. Thereafter, 5% NH_3-He was allowed at room temperature for 30 min and subsequently evacuated at 100°C for 30 min. The spectra were collected at room temperature.

UV-Raman spectra were collected using a home-built spectrometer [3]. The system was composed of a 266 nm constant-wave laser, a 25 mm diameter off-axis parabolic mirror as the light-collecting element, an edge filter to filter the Rayleigh scattered light, a spectrograph, and a UV-CCD camera produced by Andor. All spectra were calibrated by placing the main Raman peak of monocrystalline Si at 520 cm^{-1}. To ensure optical throughput, the slit width was set at 150 μm, resulting in a spectral resolution of ~7 cm^{-1}. For most experiments, the laser power at the sample was kept below 2 mW to prevent burning effects. The typical accumulation time per spectrum was ~30 s.

Structured illumination microscopy (SIM) was carried out on a Nikon N-SIM super-resolution microscopy system with a motorized inverted microscopy ECLIPSE Ti2-E, a×100/numerical aperture 1.49 oil-immersion total internal reflection fluorescence objective lens (CFI HP) and an ORCA-Flash 4.0 sCMOS camera (Hamamatsu Photonics K.K.) [4,5]. The wavelengths of illumination and emission detection of SIM were 405 (detection at 435–485 nm), 488 (detection at 500–545 nm), 561 (detection at 570–640 nm), and 640 nm (detection at 663–738 nm), respectively. The software NIS-Elements Ar and N-SIM Analysis were used to analyze the collected images and to computationally reconstruct the super-resolution image.

The uptake rate of ethane, propane and n-butane were measured by an intelligent gravimetric analyzer (IGA100, Hiden Analytical). Typically, 30.0 mg sample was evacuated below 10^{-6} Pa at 450°C prior to the sorption measurement. The adsorption kinetics curve of ethane, propane and n-butane were recorded at 30°C (0→2mbar). High purity ethane (99.999%), propane (99.995%) and n-butane (99.99%) were purchased from Dalian Special Gases Co., Ltd.

The adsorption kinetics were analyzed according to Fick's second law of diffusion [6].

$$\frac{\partial C}{\partial t} = D\frac{\partial^2 C}{\partial x^2} \qquad \textit{Eq. S1}$$

For short time:

$$\frac{m_t}{m_\infty} \approx \frac{6}{\sqrt{\pi}}\sqrt{\frac{Dt}{r^2}} \qquad \textit{Eq. S2}$$

Where m_t/m_∞ is the normalized loading, D is diffusion coefficient, t is diffusion time, and r represents characteristic diffusion length. The SAPO-17 zeolite crystals were treated as spherical adsorbent particle, and the equivalent radius r = 2.5 μm.

Catalytic reaction tests

Syngas conversion was performed in a continuous-flow fixed-bed stainless steel reactor furnished with a quartz lining. Typically, 420 mg catalyst (40-60 mesh) with $ZnCrO_x$/SAPO-17 = 1/1 (mass ratio) was used. Syngas contained 5% Ar as the internal standard for online gas chromatography (GC) analysis. Reaction was carried out under conditions of H_2/CO = 2.5, 4.0 MPa, 400°C and 5000 $mL \cdot g_{cat}^{-1} \cdot h^{-1}$ unless otherwise stated. Products were analyzed by an online GC (Agilent 7890B) equipped with a TCD and an FID. Hayesep Q and 5 Å molecular-sieve-packed columns were connected to the TCD while HP-FFAP and HP-AL/S capillary columns were connected to the FID. Oxygen-containing compounds and hydrocarbons up to C_{17} were analyzed by the FID, while CO, CO_2, CH_4, C_2H_4, and C_2H_6 were analyzed by the TCD. CH_4 and C_2H_4 were taken as a reference bridge between the FID and TCD.

CO conversion was calculated on a carbon atom basis using the following equation

$$Conv_{CO} = \frac{CO_{inlet} - CO_{outlet}}{CO_{inlet}} \times 100\% \qquad \textit{Eq. S3}$$

Where CO_{inlet} and CO_{outlet} represent moles of CO at the inlet and outlet, respectively.

CO_2 selectivity based on C atom ($SelCO_2$) was calculated according to:

$$Sel_{CO_2} = \frac{CO_{2\,outlet}}{CO_{inlet} - CO_{outlet}} \times 100\% \qquad \textit{Eq. S4}$$

Where $CO_{2\,outlet}$ denotes moles of CO_2 at the outlet.

The selectivity of individual hydrocarbon C_nH_m ($SelC_nH_m$) among hydrocarbons was obtained according to

$$Sel_{C_nH_m} = \frac{nC_nH_{m\,outlet}}{\Sigma_1^n nC_nH_{m\,outlet}} \times 100\% \qquad \textit{Eq. S5}$$

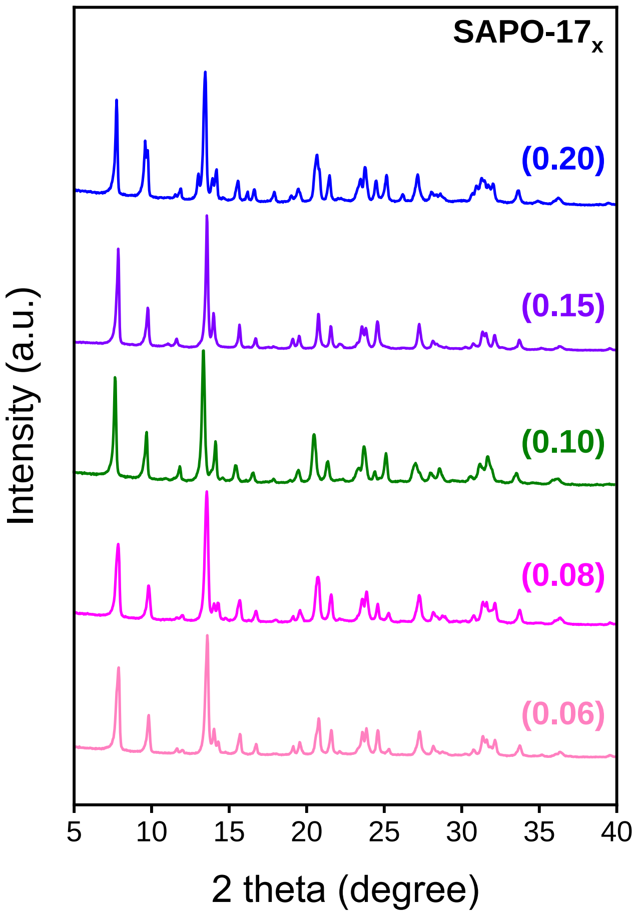

Figure S1. XRD patterns of SAPO-17$_x$ with varying Si/Al ratio.

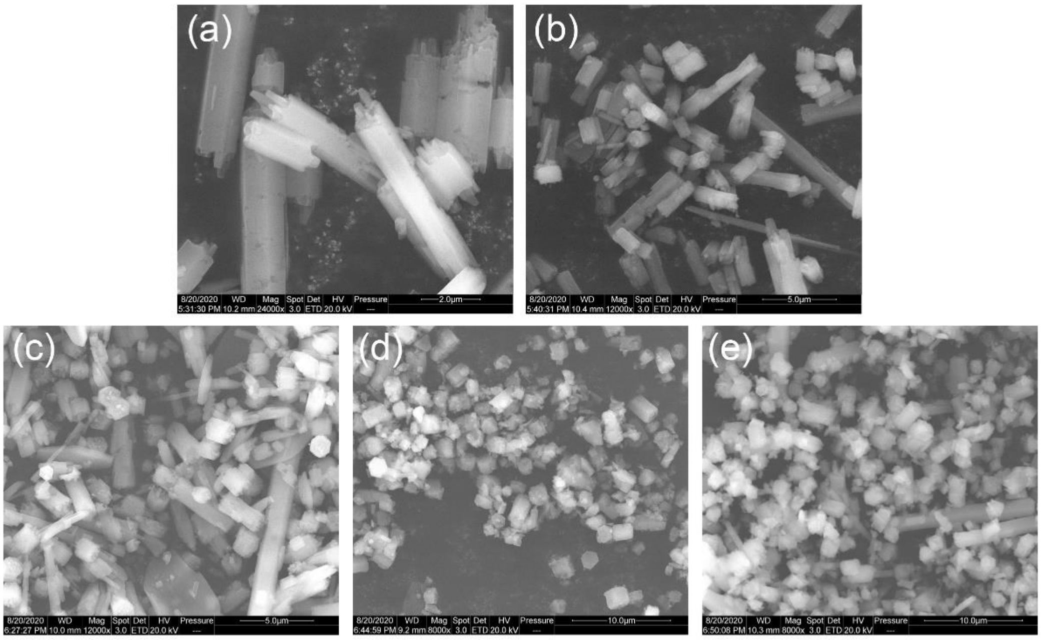

Figure S2. SEM images of SAPO-17$_x$ samples with varying Si/Al ratio. (a) 0.06; (b) 0.08; (c) 0.10; (d) 0.15; (e) 0.20.

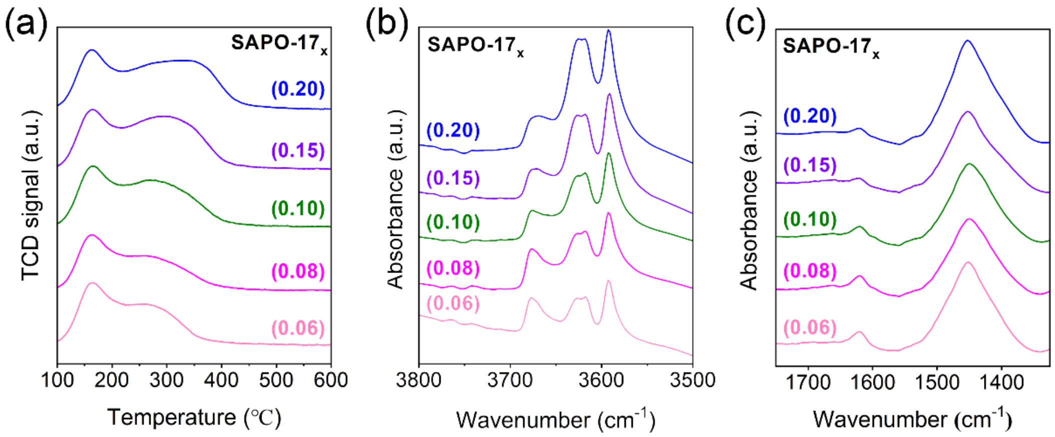

Figure S3. Acidity properties of SAPO-17$_x$. (a) NH_3-TPD profiles; (b) IR spectra of the hydroxyl stretching vibration; (c) IR differential spectra of SAPO-17$_x$ upon NH_3 adsorption.

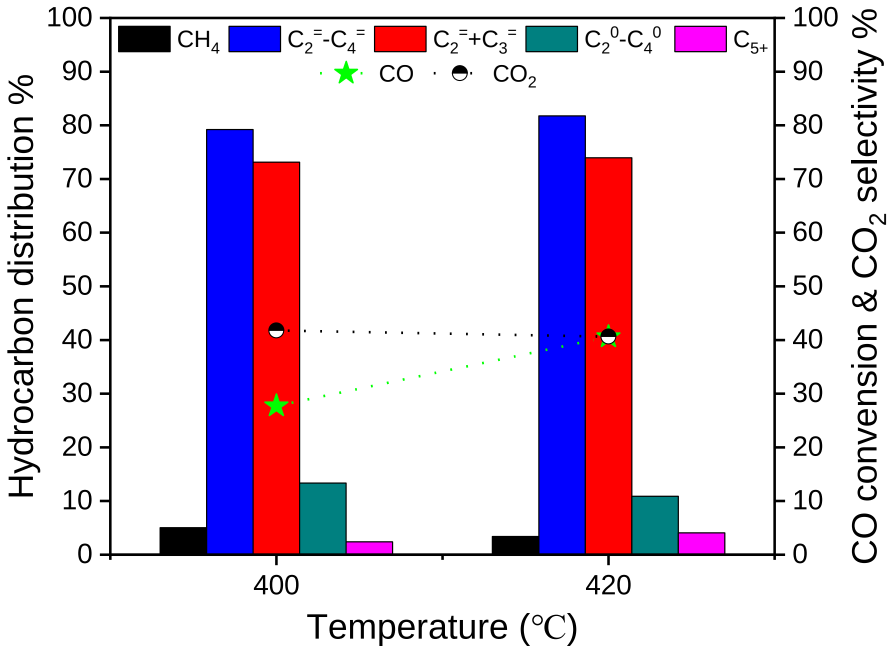

Figure S4. Effect of temperature on the catalytic performance of syngas conversion over $ZnCrO_x$-SAPO-$17_{0.10}$. Reaction conditions: $H_2/CO = 2.5$, 4.0 MPa, $ZnCrO_x$/SAPO-17 = 1/1 (mass ratio), 5000 mL·g^{-1}·h^{-1}.

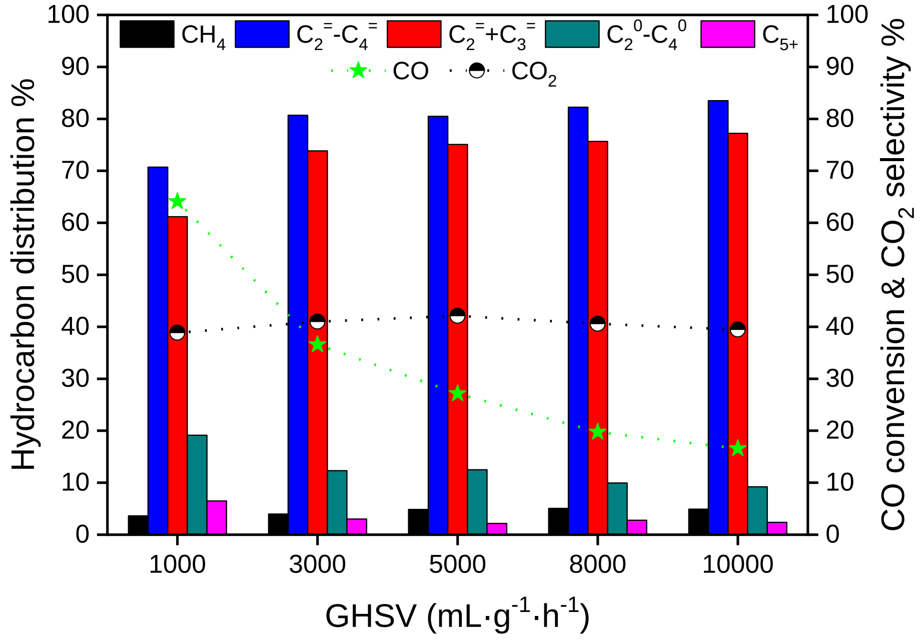

Figure S5. Effect of gas hourly space velocities (GHSV) on the catalytic performance of syngas conversion over $ZnCrO_x$-SAPO-$17_{0.10}$. Reaction conditions: 400℃, H_2/CO = 2.5, 4.0 MPa, $ZnCrO_x$/SAPO-17 = 1/1 (mass ratio).

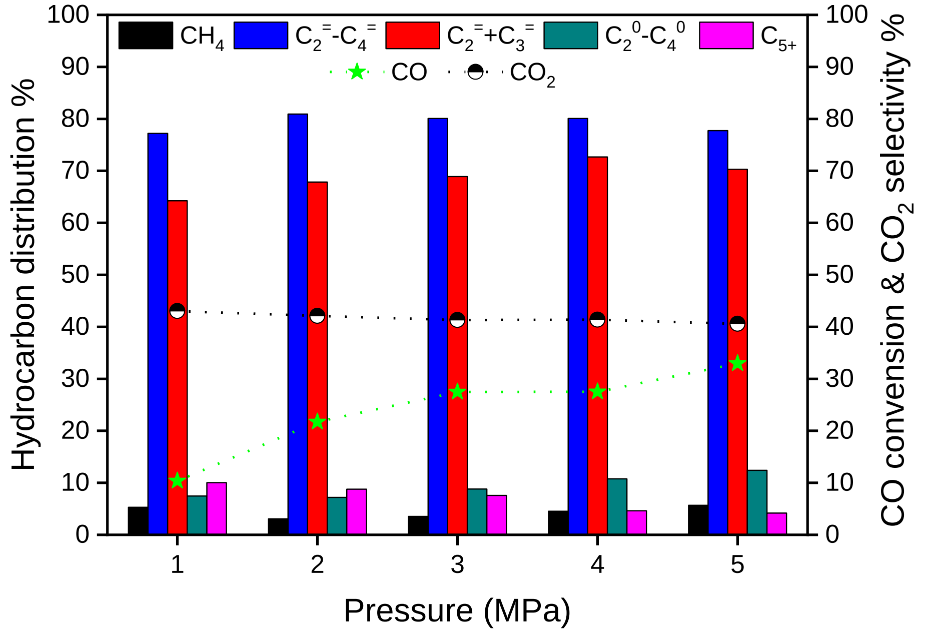

Figure S6. Effect of pressure on the catalytic performance of syngas conversion over $ZnCrO_x$-SAPO-$17_{0.10}$. Reaction conditions: 400 ℃, $H_2/CO = 2.5$, $ZnCrO_x$/SAPO-17 = 1/1 (mass ratio), 5000 $mL \cdot g^{-1} \cdot h^{-1}$.

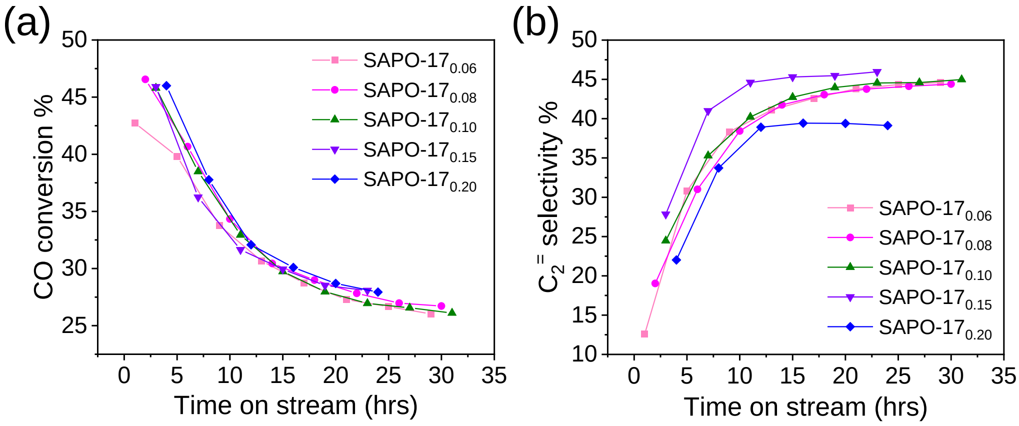

Figure S7. Syngas conversion performance over ZnCrO$_x$-SAPO-17$_x$ with various Si/Al ratio. (a) CO conversion and (b) $C_2^=$ selectivity evolution with time on stream.

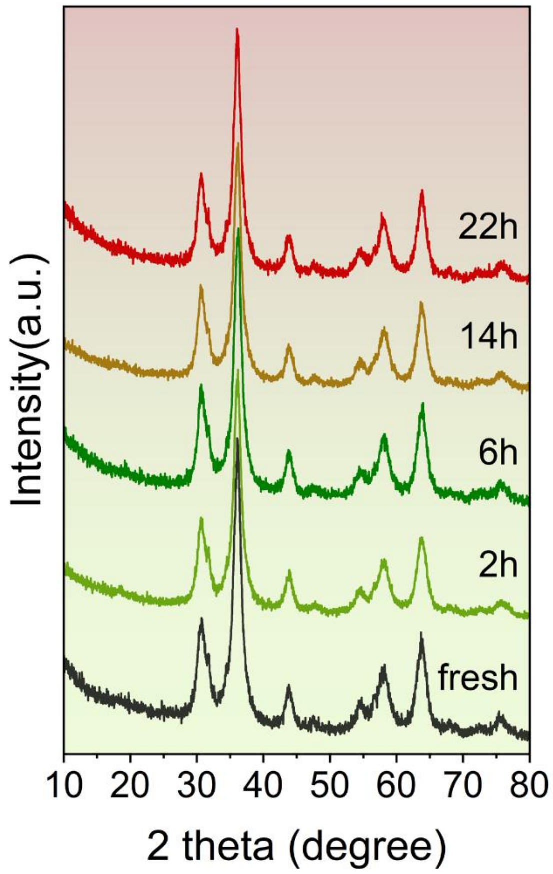

Figure S8. XRD patterns of fresh $ZnCrO_x$ and $ZnCrO_x$ after reaction for 2, 6, 14 and 22 h, respectively.

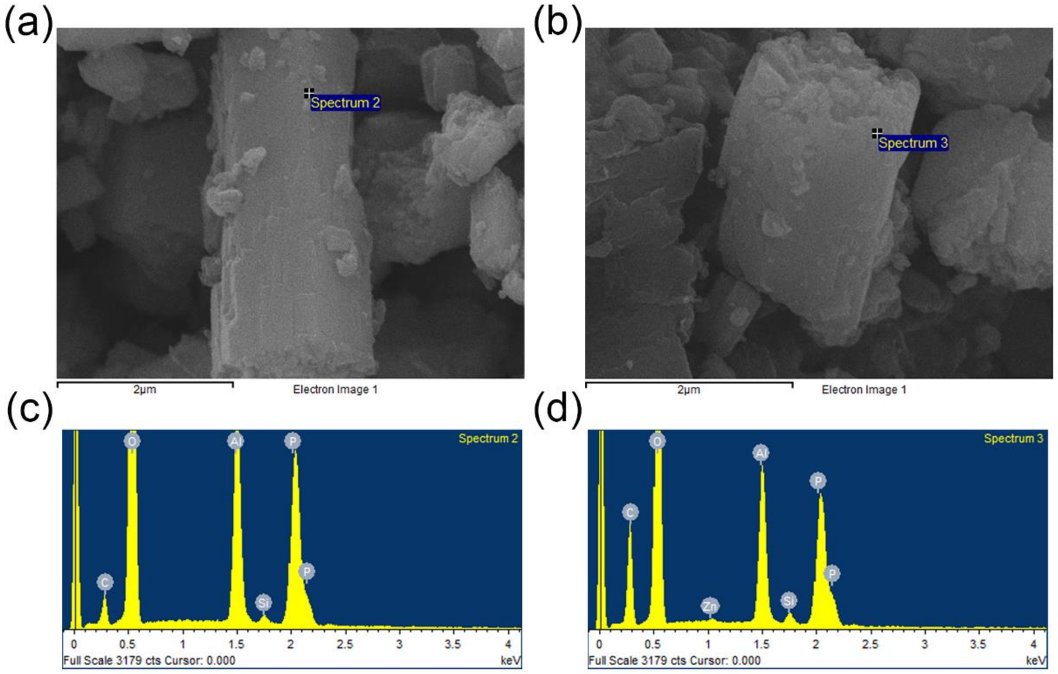

Figure S9. SEM images and EDS analysis of SAPO-17 after reaction for 2 h (a and c) and 22 h (b and d).

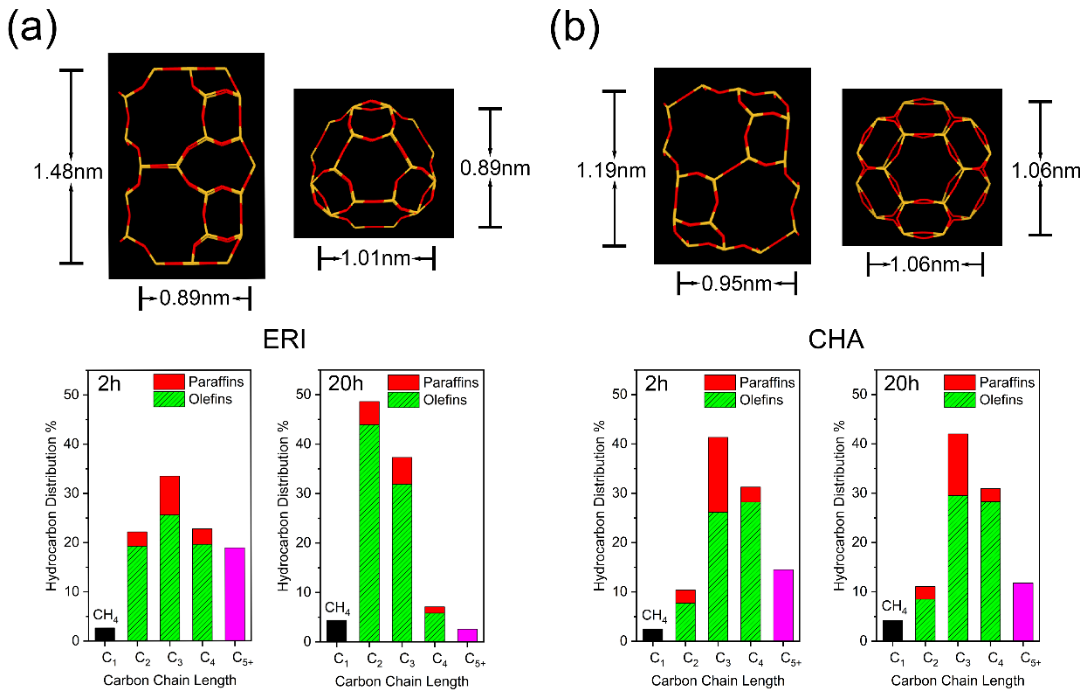

Figure S10. Comparison of the hydrocarbon distributions in syngas conversion over different composite catalysts at 2h and 20h. (a) $ZnCrO_x$-SAPO-17. (b) $ZnCrO_x$-SAPO-34. Reaction conditions: 400℃, $H_2/CO = 2.5$, 4.0 MPa, $ZnCrO_x$/SAPO = 1/1 (mass ratio), 5000 $mL\cdot g^{-1}\cdot h^{-1}$.

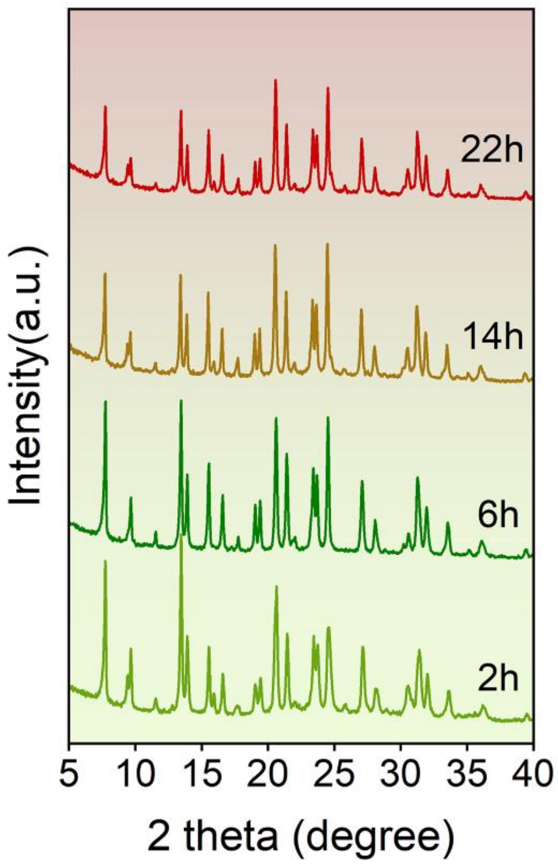

Figure S11. XRD patterns of SAPO-17 after reaction for 2, 6, 14 and 22 h, respectively.

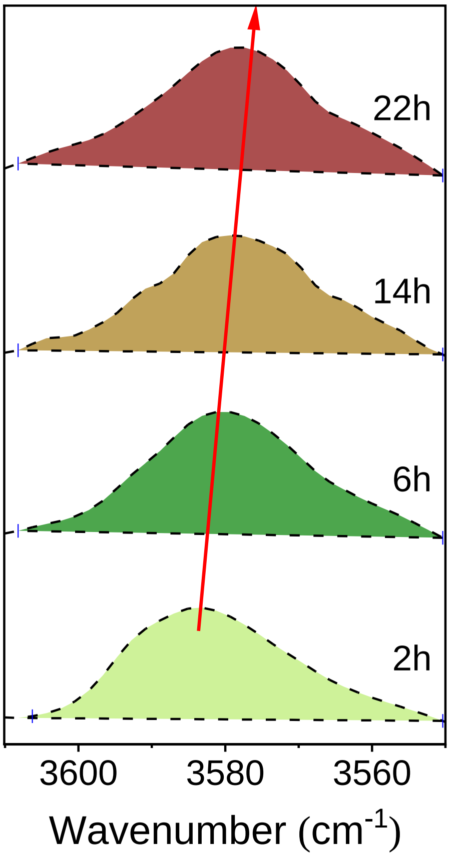

Figure S12. IR spectra showing the stretching vibration of Si-OH-Al for SAPO-17 after reaction for 2, 6, 14 and 22 h, respectively.

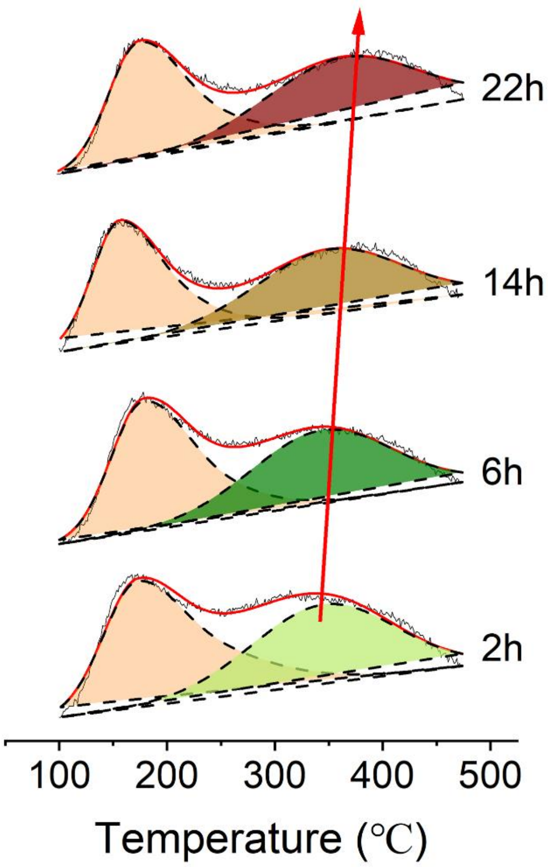

Figure S13. NH_3-TPD profiles of SAPO-17 after reaction for 2, 6, 14 and 22 h, respectively.

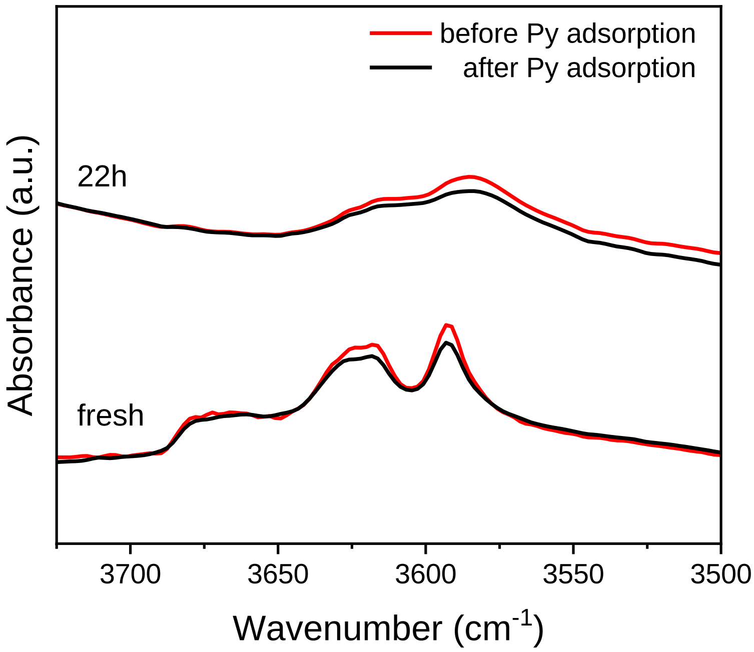

Figure S14. Pyridine adsorption IR spectra of the hydroxyl stretching vibration region of fresh SAPO-17 and SAPO-17 after reaction for 22h.

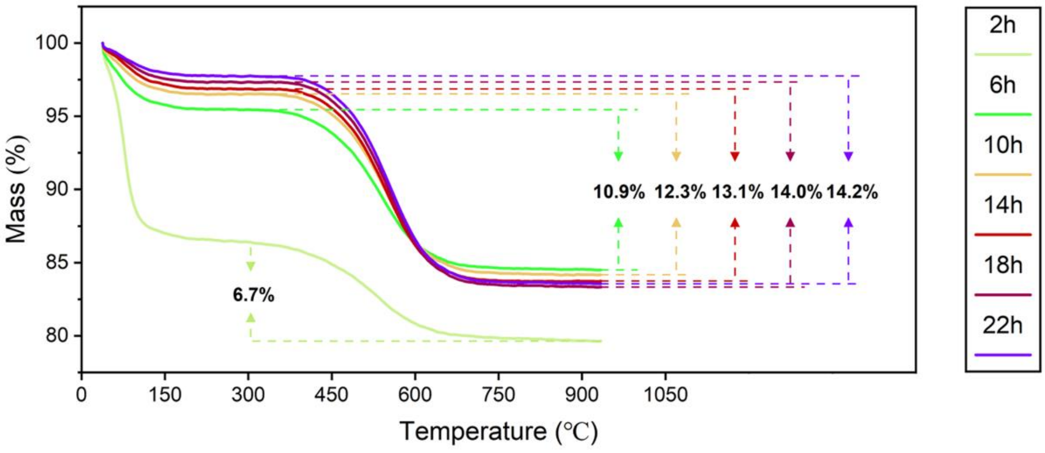

Figure S15. TG analysis of the SAPO-17 samples after reaction for various time.

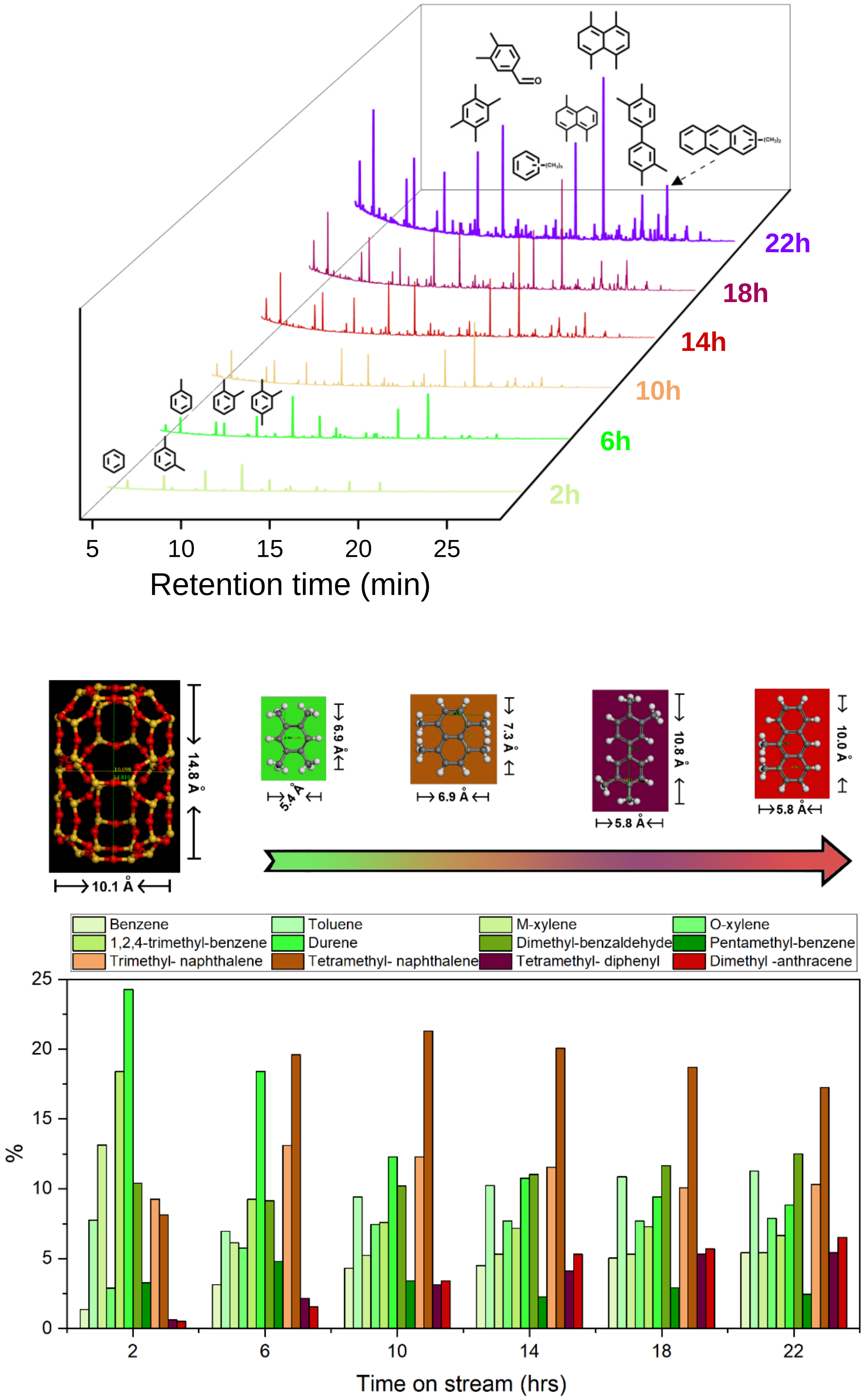

Figure S16. GC-MS analysis of the confined carbonaceous species in the SAPO-17 cage after reaction for 2, 6, 10, 14, 18 and 22 h, respectively.

Additional discussion:

The dual-cycle mechanism is widely accepted for MTH reaction. Ethylene and propylene are produced in a similar quantity in the classical aromatic cycle while propylene and higher hydrocarbons dominate following olefinic cycle. Following the aromatic cycle, Haw and co-workers further showed that ethylene would dominate the products if the aromatic compounds in H-SAPO-34 cage contain less substituent groups e.g., trimethylbenzene and tetramethylbenzene whereas propylene would be favored from pentamethylbenzene and hexamethylbenzene [7]. A similar phenomenon was reported by Bjørgen and coworkers for the aromatic cycle over H-ZSM-5. In addition, pentamethylbenzene and hexamethylbenzene were reported in the cages of H-beta, which also favored formation of propylene [8].

In this study, the aromatics species are dominated in SAPO-17 cage during the entire induction period of syngas conversion. Trimethylbenzene, tetramethylbenzene and methylnaphthalene predominate during the first 10 h of the induction period (Supplementary Fig. S10). Thereafter, tetramethyl-diphenyl and dimethyl-anthracene increase while trimethylbenzene and tetramethylbenzene decrease gradually. According to the aromatic cycle of MTH, ethylene should be more favored during the first 10 h and then propylene selectivity should increase while ethylene selectivity should decrease. However, Figure 1B shows an opposite trend. Therefore, the selectivity change during the induction period of syngas conversion is not controlled by the dual-cycle mechanism.

The difference in mechanism between OXZEO and MTH has also been previously observed. For instance, in our previous study on $ZnCrO_x$-SAPO-18 bifunctional OXZEO catalyst, a stronger acid strength and higher acid density of SAPO-18 leads to a higher C_3/C_2 ratio in the products [9]. By contrast, a stronger acid strength and higher acid density benefit the aromatic cycle, giving a lower C_3/C_2 ratio in MTH catalyzed by various zeolites, e.g., SAPO-34, SSZ-13, and ZSM-5 [10-12].

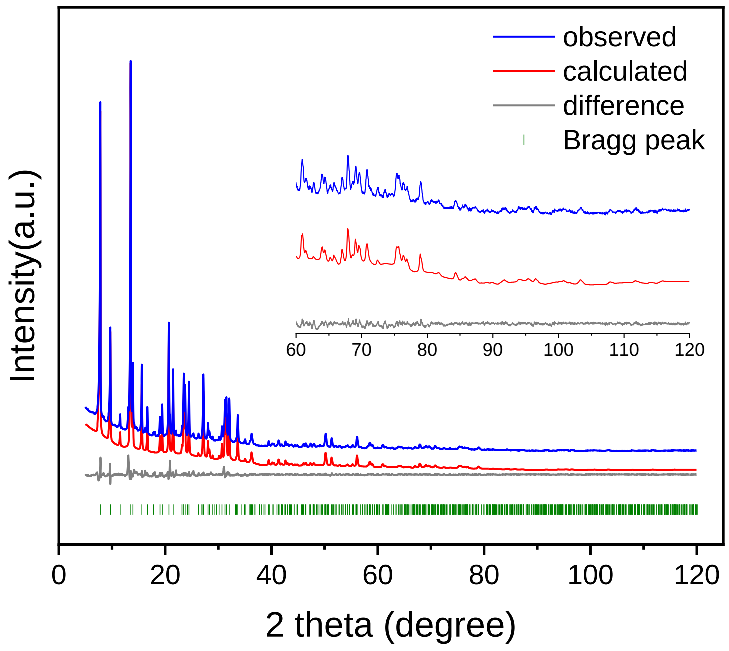

Figure S17. Rietveld refinement plots of the fresh SAPO-17. The measured, calculated, difference curves and Bragg peak are in blue, red, black and green, respectively. The refinement typically converged to Rwp-background=5.208%.

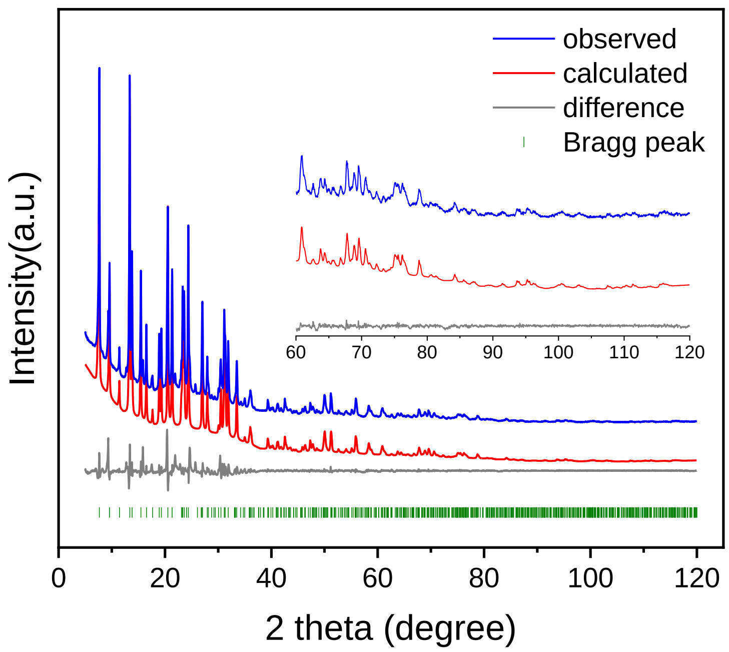

Figure S18. Rietveld refinement plots of SAPO-17 after syngas conversion for 2h. The observed, calculated, difference curves and Bragg peak are in blue, red, black and green, respectively. The refinement typically converged to Rwp-background=5.490%.

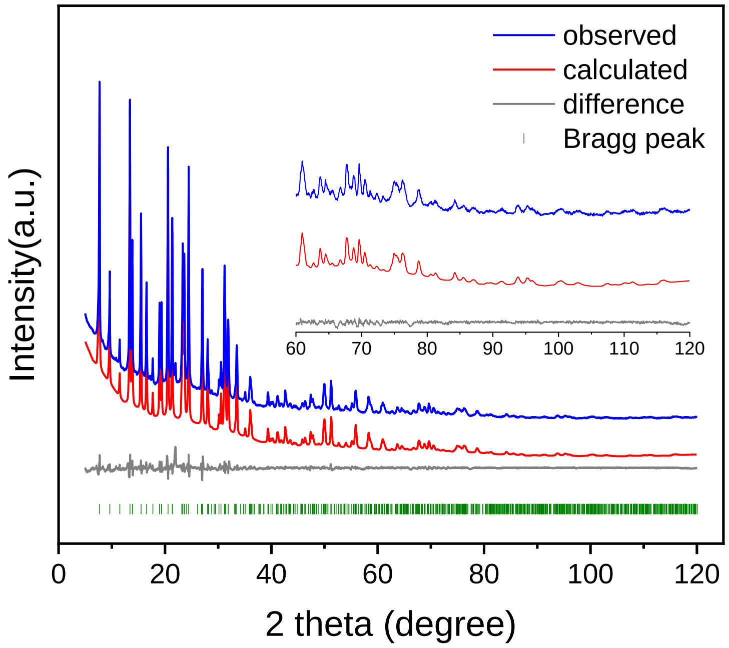

Figure S19. Rietveld refinement plots of SAPO-17 after syngas conversion for 6h. The observed, calculated, difference curves and Bragg peak are in blue, red, black and green, respectively. The refinement typically converged to Rwp-background=3.409%.

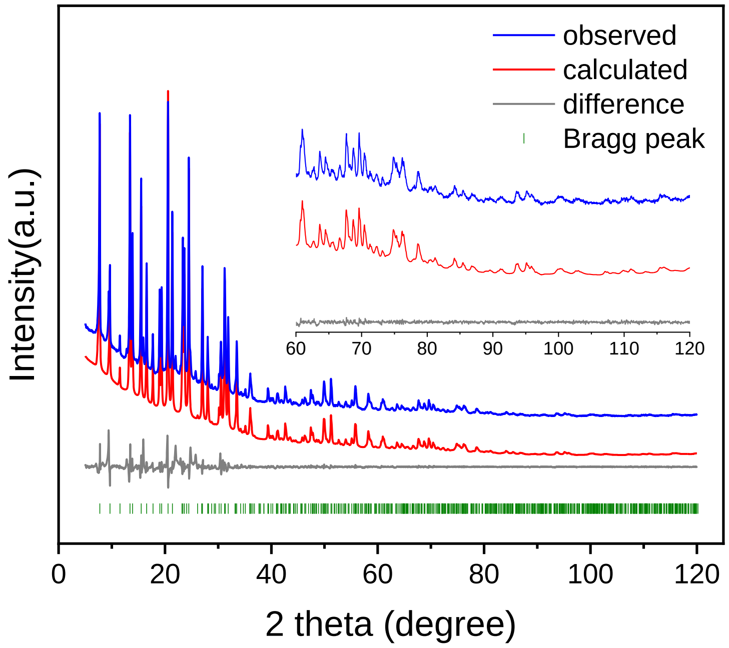

Figure S20. Rietveld refinement plots of SAPO-17 after syngas conversion for 14h. The observed, calculated, difference curves and Bragg peak are in blue, red, black and green, respectively. The refinement typically converged to Rwp-background=4.455%.

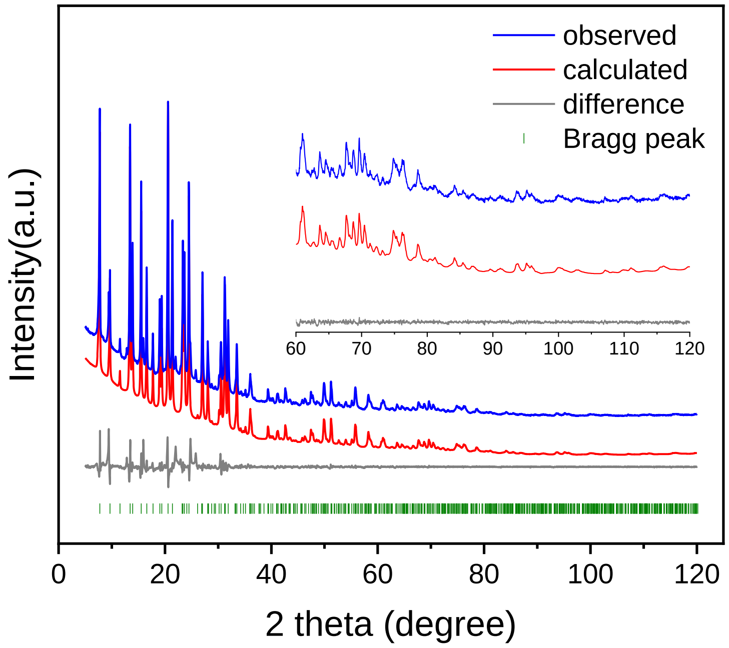

Figure S21. Rietveld refinement plots of SAPO-17 after syngas conversion for 22h. The observed, calculated, difference curves and Bragg peak are in blue, red, black and green, respectively. The refinement typically converged to Rwp-background=4.567%.

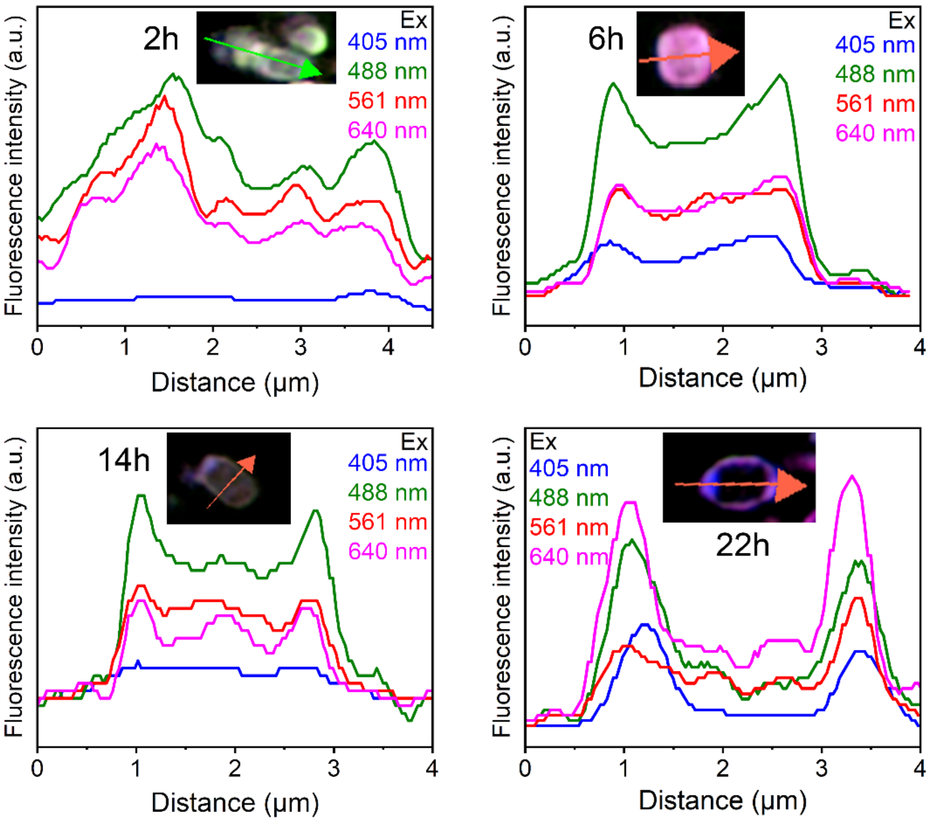

Figure S22. The spatiotemporal distribution of carbonaceous species over SAPO-17 after reaction for different TOS, obtained from SIM. The overlap of four profiles with different laser excitations of 405 nm (detected at 435–485 nm, blue curve), 488 nm (detected at 500–545 nm, green curve), 561 nm (detected at 570–640 nm, red curve), 640 nm (detected at 663–738 nm, pink curve).

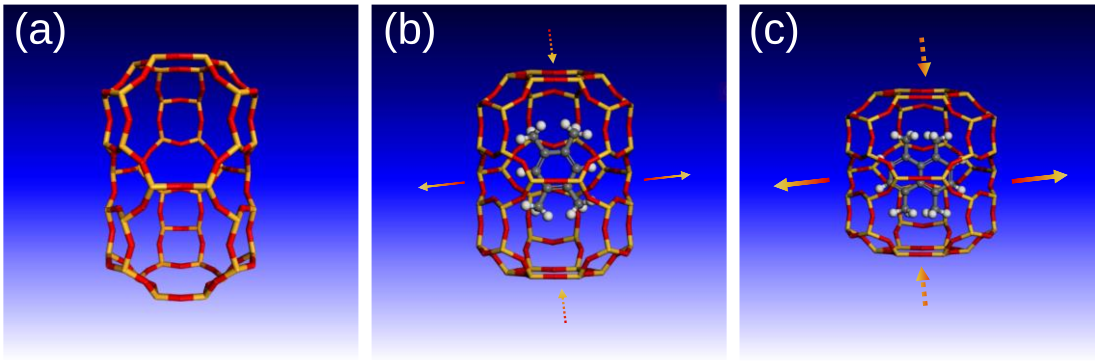

Figure S23. Scheme for the lattice distortion due to confined carbonaceous species. (a) Empty ERI-cage. (b) Durene confined inside ERI-cage. (c) Tetramethyl-naphthalene confined inside ERI-cage.

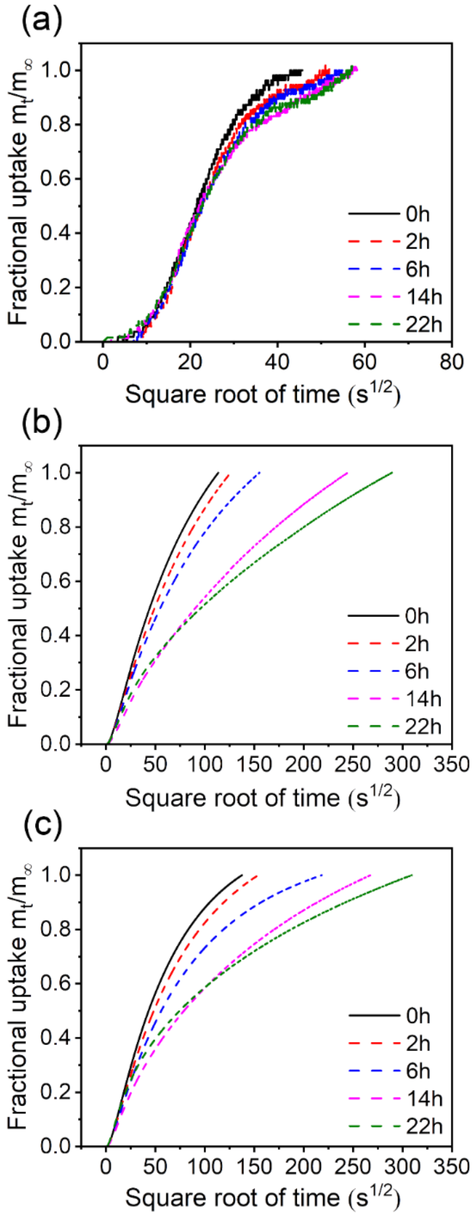

Figure S24. Diffusion of guest molecules in different SAPO-17 samples, analyzed by IGA at 30°C (0→2 mbar). (a) Ethane. (b) Propane. (c) n-butane.

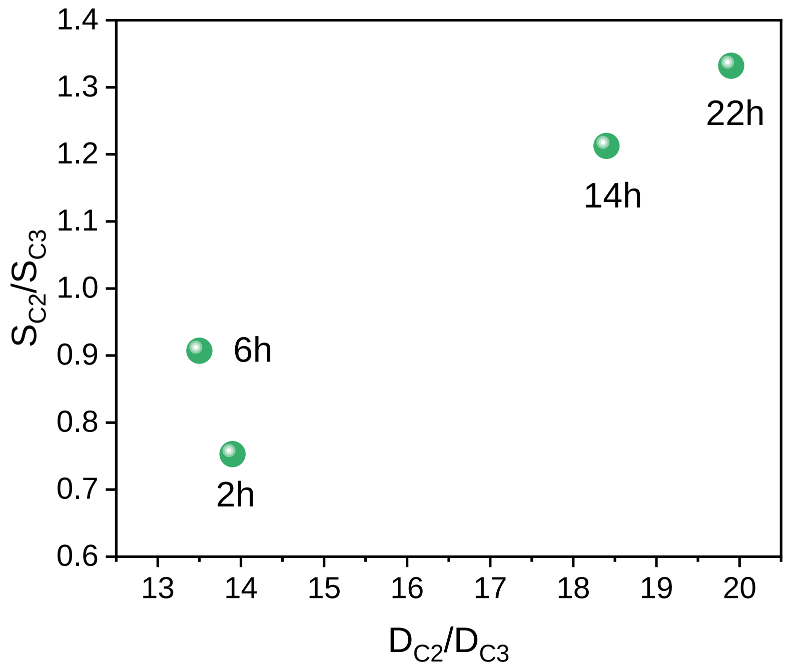

Figure S25. Relation between the selectivity S_{C2}/S_{C3} and the diffusion of D_{C2}/D_{C3}. Reaction conditions: 400℃, H_2/CO = 2.5, 4.0 MPa, $ZnCrO_x$/SAPO-17 = 1/1 (mass ratio), 5000 mL·g^{-1}·h^{-1}.

Table S1. Properties of SAPO-17 with varying Si/Al ratio.

Sample	SiO_2/Al_2O_3		Surface area (m^2/g)			Pore volume (cm^3/g)	
	x	y[a]	S_{micro}	S_{ext}	S_{total}	V_{micro}	V_{total}
SAPO-$17_{0.06}$	0.06	0.0543	538.3	54.9	593.2	0.210	0.310
SAPO-$17_{0.08}$	0.08	0.0747	534.9	51.2	586.1	0.210	0.325
SAPO-$17_{0.10}$	0.10	0.0943	557.5	49.5	607.0	0.218	0.321
SAPO-$17_{0.15}$	0.15	0.1403	557.4	45.9	603.3	0.218	0.312
SAPO-$17_{0.20}$	0.20	0.1933	572.9	33.2	606.1	0.223	0.283

[a] Measured by XRF.

Table S2. The crystal size of fresh $ZnCrO_x$ and $ZnCrO_x$ after reaction for 2, 6, 14 and 22 h, respectively.

Sample	crystal size (nm)
$ZnCrO_x$	6.1
$ZnCrO_x$-2h	6.5
$ZnCrO_x$-6h	6.5
$ZnCrO_x$-14h	6.6
$ZnCrO_x$-22h	6.6

Table S3. Projected size of the SAPO-17 cage and the carbonaceous species.

**Structure**		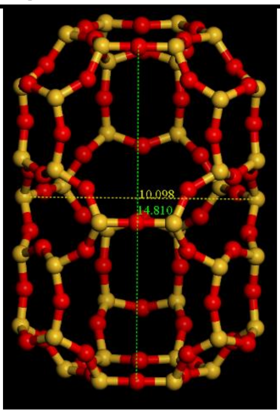	
**Size (Å)**		**14.8*10.1**	
**Structure**	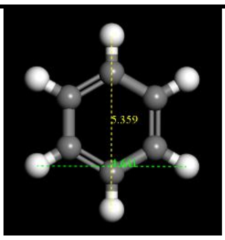	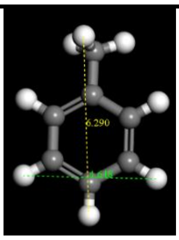	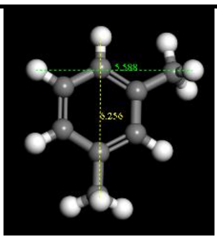
**Size (Å)**	**5.4*4.6**	**6.3*4.6**	**6.3*5.6**
**Structure**	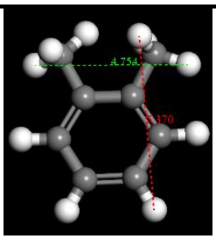	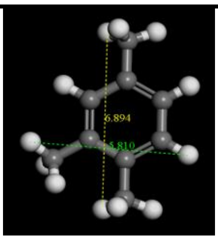	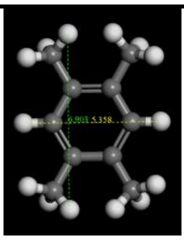
**Size (Å)**	**5.4*4.8**	**6.9*5.8**	**6.9*5.4**
**Structure**	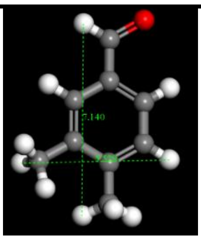	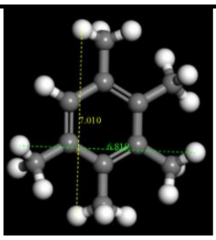	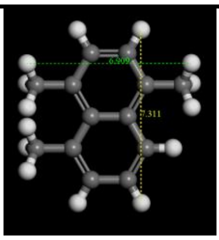
**Size (Å)**	**7.1*5.5**	**7.0*6.8**	**7.3*6.9**
**Structure**	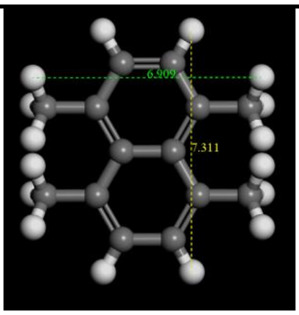	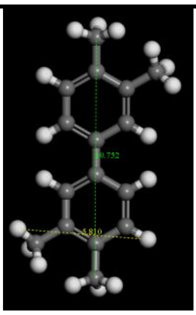	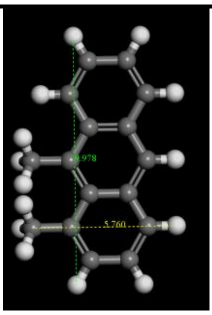
**Size (Å)**	**7.3*6.9**	**10.8*5.8**	**10.0*5.8**

Table S4. Projected size of the SAPO-17 cage and the carbonaceous species considering van der Waals radius of 2.5 Å.

molecule	**Size (Å)**
	A_i
ERI-cage	14.8*10.1
Benzene	7.9*7.1
Toluene	8.8*7.1
m-xylene	8.8*8.1
o-xylene	7.9*7.3
1,2,4-trimethyl-benzene	9.4*8.3
Durene	9.4*7.9
Dimethyl-benzaldehyde	9.6*8.0
Pentamethyl-benzene	9.5*9.3
Trimethyl-naphthalene	9.8*9.4
Tetramethyl-naphthalene	9.8*9.4
Tetramethyl-diphenyl	13.3*8.3
Dimethyl-anthracene	12.5*8.3

Table S5. The diffusion coefficients (D) of ethane, propane and n-butane over (SAPO-17)-xh at 30°C under 2 mbar corresponding to the data in Supplementary Fig. S24.

Samples	ethane	propane	n-butane	D_{C2}/D_{C4}	D_{C2}/D_{C3}
	D_{C2} $\times 10^{-12}$ cm^2/s	D_{C3} $\times 10^{-13}$ cm^2/s	D_{C4} $\times 10^{-13}$ cm^2/s		
SAPO-17-0h	11.2	8.6	8.4	13.4	13.0
SAPO-17-2h	9.2	6.6	6.0	15.4	13.9
SAPO-17-6h	7.0	5.2	4.0	17.5	13.5
SAPO-17-14h	4.3	2.4	2.0	21.5	18.4
SAPO-17-22h	6.4	3.2	2.5	25.3	19.9

REFERENCES

1. Jiao F, Li JJ and Pan XL *et al.* Selective conversion of syngas to light olefins. *Science*. 2016; **351**: 1065-1068.
2. Zhong SL, Song SC and Wang B *et al.* Fast preparation of ERI-structure AlPO-17 and SAPO-17 in the presences of isomorphous and heterogeneous seeds. *Micropor Mesopor Mat* 2018; **263**: 11-20.
3. An HY, Zhang F and Guan ZH *et al.* Investigating the coke formation mechanism of H-ZSM-5 during methanol dehydration using operando UV-Raman spectroscopy. *ACS Catalysis* 2018; **8**: 9207-9215.
4. Wong YC, Ysselstein Dand and Krainc D. Mitochondria-lysosome contacts regulate mitochondrial fission via RAB7 GTP hydrolysis. *Nature* 2018; **554**: 382-386.
5. Qi QK, Chi WJ and Li YY *et al.* A H-bond strategy to develop acid-resistant photoswitchable rhodamine spirolactams for super-resolution single-molecule localization microscopy. *Chem Sci* 2019; **10**: 4914-4922.
6. Karger J and Ruthven DM. Diffusion in nanoporous materials: fundamental principles, insights and challenges. *New J Chem* 2016; **40**: 4027-4048.
7. Song WG, Fu H and Haw JF. Supramolecular origins of product selectivity for methanol-to-olefin catalysis on HSAPO-34. *Journal of the American Chemical Society* 2001; **123**: 4749-4754.
8. Svelle S, Olsbye U and Joensen F *et al.* Conversion of methanol to alkenes over medium- and large-pore acidic zeolites: Steric manipulation of the reaction intermediates governs the ethene/propene product selectivity. *J Phys Chem C* 2007; **111**: 17981-17984.
9. Li G, Jiao F and Pan XL *et al.* Role of SAPO-18 acidity in direct syngas conversion to light olefins. *ACS Catalysis* 2020; **10**: 12370-12375.
10. Bleken F, Bjorgen M and Palumbo L *et al.* The effect of acid strength on the conversion of methanol to olefins over acidic microporous catalysts with the CHA topology. *Top Catal* 2009; **52**: 218-228.
11. Li ZB, Martinez-Triguero J and Concepcion P *et al.* Methanol to olefins: activity and stability of nanosized SAPO-34 molecular sieves and control of selectivity by silicon distribution. *Phys Chem Chem Phys* 2013; **15**: 14670-14680.
12. Yarulina I, De Wispelaere K and Bailleul S *et al.* Structure-performance descriptors and the role of Lewis acidity in the methanol-to-propylene process. *Nat Chem* 2018; **10**: 804-812.
